# Ultrafiltration is better than diuretic therapy for volume-overloaded acute heart failure patients: a meta-analysis

**DOI:** 10.1007/s10741-020-10057-7

**Published:** 2020-11-26

**Authors:** Bastian Wobbe, Juliane Wagner, Dorottya Kata Szabó, Ildikó Rostás, Nelli Farkas, András Garami, Márta Balaskó, Petra Hartmann, Margit Solymár, Judit Tenk, Máté Ottóffy, Arnold Nagy, Tamás Habon, Péter Hegyi, László Czopf

**Affiliations:** 1 Division of Cardiology, 1st Department of Medicine, University of Pécs Medical School, Pécs, Hungary; 2 Institute for Translational Medicine, University of Pécs Medical School, Pécs, Hungary; 3 Department for Pediatrics, University of Pécs Medical School, Pécs, Hungary; 4 Institute of Surgical Research, University of Szeged, Szeged, Hungary; 5 Division of Cardiology, 1st Department of Medicine, University of Pécs Medical School, Ifjúság u. 13., Pécs, H-7624 Hungary

**Keywords:** Acute heart failure, Ultrafiltration, Diuretics, Meta-analysis

## Abstract

Studies on the effectiveness of ultrafiltration (UF) in patients hospitalized with acute decompensated heart failure (ADHF) have led to heterogeneous study outcomes. This meta-analysis aimed to assess the impact of UF therapy in ADHF patients. We searched the medical literature to identify well-designed studies comparing UF with the usual diuretic therapy in this setting. Systematic evaluation of 8 randomized controlled trials enrolling 801 participants showed greater fluid removal (difference in means 1372.5 mL, 95% CI 849.6 to 1895.4 mL; *p* < 0.001), weight loss (difference in means 1.592 kg, 95% CI 1.039 to 2.144 kg; *p* < 0.001) and lower incidences of worsening heart failure (OR 0.63, 95% CI 0.43 to 0.94, *p* = 0.022) and rehospitalization for heart failure (OR 0.54, 95% CI 0.36 to 0.82, *p* = 0.003) without a difference in renal impairment (OR 1.386, 95% CI 0.870 to 2.209; *p* = 0.169) or all-cause mortality (OR 1.13, 95% CI 0.75 to 1.71, *p* = 0.546). UF increases fluid removal and weight loss and reduces rehospitalization and the risk of worsening heart failure in congestive patients, suggesting ultrafiltration as a safe and effective treatment option for volume-overloaded heart failure patients.

## Introduction

Heart failure (HF) is a clinical syndrome characterized by a group of symptoms (e.g., dyspnea, fatigue, and ankle swelling) and signs (e.g., pulmonary crackles and peripheral edema) [1]. Acute decompensation is a common complication in patients with chronic HF.

Ultrafiltration (UF) is an upcoming treatment alternative for patients hospitalized for acute decompensated heart failure (ADHF). Fluid overload remains the main cause of heart failure hospitalization and is driven by sodium and water retention.[2] Thus, decongestion is one of the primary targets of therapy.

Mostly, decongestion has been accomplished through diuretic treatment. While 88% of affected patients are treated with diuretics, many show suboptimal responses, and hospital readmission rates remain high (25% within 30 days) [3, 4]. Concerns related to the safety and efficacy of diuretic treatment have also been raised. Furthermore, randomized controlled trials (RCTs) investigating the effects of diuretics on mortality and morbidity are lacking.[1] Chronic and combined use of diuretics might lead to adverse effects such as diuretic resistance, electrolyte imbalances, and deteriorating renal function.[5] Such concerns have led to a growing interest in the development of novel therapeutic approaches for decongestion.

Ultrafiltration, a type of renal replacement therapy, is an invasive procedure that creates a transmembrane pressure gradient driving plasma fluid across a semipermeable membrane. This technique can be used as an alternative to diuretic treatment for the removal of excess fluid in volume-overloaded HF patients. Through technical improvements, UF devices have decreased in size, and their handling was simplified, which may contribute to the more extensive use of this therapeutic procedure in the future. Current guidelines promote the limited use of UF as a second-line treatment option for patients failing to respond to diuretic therapy or for those developing diuretic resistance [1, 6].

However, the results of Costanzo et al. convincingly proposed the use of UF as an initial treatment over diuretics in ADHF patients with volume overload [7, 8]. Costanzo et al. reported greater weight loss and fluid removal in the group treated with UF compared to the group treated with diuretics, as well as a 53% decreased risk of rehospitalization after 6 months.

A number of small RCTs have investigated the influence of UF treatment compared to usual care therapy so far, but the results remain heterogeneous [9–11].

We performed this meta-analysis to investigate the possible advantages of UF compared to diuretic treatment regarding fluid removal, weight loss, rehospitalization for HF, and all-cause mortality. Furthermore, incidences of adverse events were evaluated to highlight possible risks associated with this therapy.

## Methods

### Protocol and structure

This meta-analysis is reported based on the Preferred Reporting Items for Systematic Reviews and Meta-Analyses (PRISMA) Statement [12].

### Search strategy

We searched MEDLINE (via PubMed), Embase, and the Cochrane Central Register of Controlled Trials (CENTRAL) to identify RCTs that compared ultrafiltration to “usual care” (diuretic therapy) in patients with ADHF. The date of the search was January 20, 2018, and the search was performed using the search terms “acute heart failure,” “ultrafiltration,” “diuretic agent” (for Embase and CENTRAL), or “diuretics” (for MEDLINE) and filtered for randomized controlled human trials published in English.

### Study selection

The initial screening of the articles was based on reading the titles and abstracts of the studies retrieved by our search strategy to determine suitability. After removing duplicates, a second evaluation was performed, which included reading the full-text articles. The references of the identified articles were manually searched for further relevant publications. The selection process was performed by two independent reviewers, and any disagreements were resolved through discussion. The process of inclusion and exclusion throughout the different phases is visually displayed using the PRISMA 2009 Flow Diagram [12].

We applied the following eligibility criteria:

RCTs involving adults diagnosed with ADHF who presented signs and symptoms of congestion in which UF intervention was compared with usual care treatment using diuretic agents. For inclusion, studies needed to report one or more of the following study outcomes: fluid removal, weight loss, all-cause mortality, heart failure-related rehospitalization, or adverse events, such as worsening heart failure or renal impairment (the latter was defined as an increase in serum creatinine level, a decrease in glomerular filtration rate, renal failure, or the need for dialysis).

We excluded those studies in which data seemed to be partly or completely evaluated in another selected study as well. Retrospective studies or studies without a control group were not included in our analysis.

### Assessment of the risk of bias in included studies

The Cochrane Risk of Bias Tool for quality assessment of RCTs was used. We evaluated information concerning sequence generation, allocation concealment, blinding, outcome reporting, patient withdrawal, and other possible sources of bias.[13].

### Data extraction

The extracted data included first author, year of publication, country of the study, patient inclusion and exclusion criteria, patient characteristics (number of patients, age, sex, medication, and comorbidities including hypertension and diabetes mellitus), details of the intervention (type of UF device, UF rate, and duration of UF session), details of the control-group protocol (type, dosage and administration route of diuretics), quality indicators (randomization, blinding, patient withdrawals/dropouts, and completion of follow-up), and study outcomes such as fluid removal, weight loss, number of adverse events, all-cause mortality, rehospitalization for heart failure with time of measurements, and follow-up duration.

### Measures of treatment effect

The following primary outcomes were defined in our analysis: (i) amount of fluid removal in milliliters, (ii) number of patients rehospitalized for HF during the follow-up, and (iii) incidences of adverse events such as worsening HF and renal impairment (defined as an increase in serum creatinine level, a decrease in glomerular filtration rate, renal failure or the need for dialysis). Secondary outcomes included (i) weight loss measured in kilograms and (ii) all-cause mortality during follow-up.

### Statistical analysis

Comprehensive Meta-Analysis (Version 3) statistical software (Biostat, Inc., Engelwood, MJ, USA) was used for data analyses. In the case of binary outcomes, we calculated the pooled odds ratio (OR) and 95% confidence interval (CI), and in the case of continuous variables, we calculated the difference in means and standard error (SE). A fixed-effects model by Mantel–Haenszel was used in all cases since all the included studies were RCTs.[14] We suggested a statistically significant effect if the CI did not include 1 and the *p* value was less than 0.05. Heterogeneity was tested using Cochran’s *Q* and the *I*^2^ statistics.[13, 15] The *Q* homogeneity test statistic exceeds the upper-tail critical value of the chi-square on *k*–1 degrees of freedom. A *p* value of less than 0.1 was considered suggestive of significant heterogeneity. The *I*^2^ statistic represents the percentage of the total variability across studies. Data are presented for visual comparisons using forest plots. Publication bias was examined by visual inspection of funnel plots, in which the SE was plotted against the net change for each study.

## Results

### Results of the search and description of studies

**Fig. 1:**
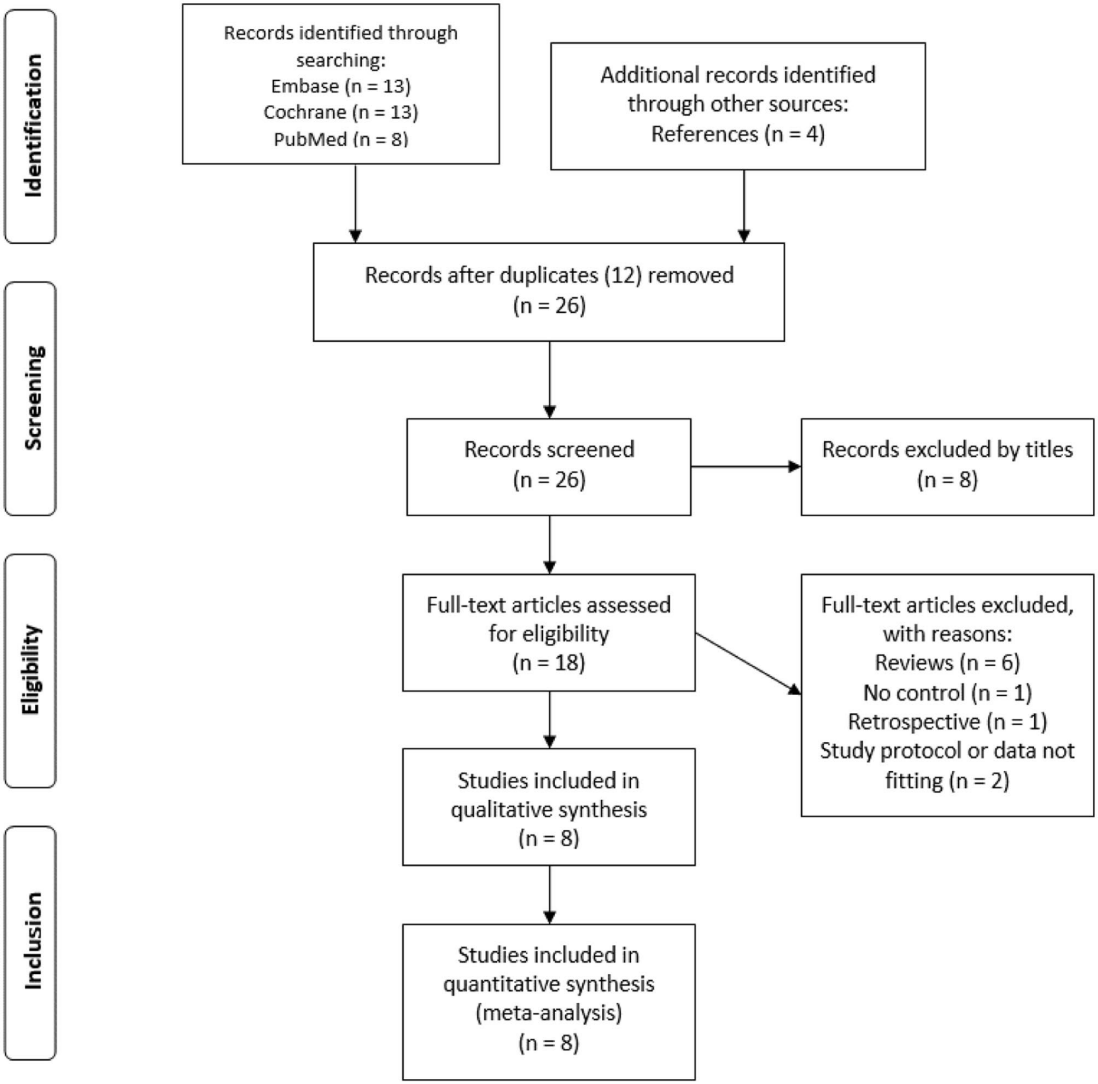
Flowchart of trial selection. Study selection flowchart according to PRISMA-P [12]

We initially identified 34 potentially relevant articles from the three different databases. After removing 12 duplicates, 8 articles were excluded by the titles. Eighteen articles were included in the full-text review. After reading the abstracts and the full texts, 10 studies were excluded: 2 studies did not provide any data on the investigated outcomes, 6 articles were reviews, one study failed to provide a control group, and one study was excluded because it was a retrospective analysis. Additionally, we searched the references manually for relevant articles at this stage of our search. We identified 4 further RCTs that were suitable for our analysis. Ultimately, 8 RCTs were included in our meta-analysis (Fig. 1).[7, 9–11, 16–19].

Table 1 shows the characteristics of the studies that were included in this meta-analysis, while the outcomes of the included studies are shown in Table 2. The 8 trials enrolled 801 participants. The sample sizes of the included studies varied, ranging from 30 to 221. The UF rate ranged from 100 to 500 mL/h, while different types of UF devices were used (Aquadex System 100, NxStage System One and PRISMA System). Loop diuretics (furosemide) were used in all trials, with a maximum dosage of up to 1446 mg/day.

**Table 1:** Characteristics of randomized controlled trials

First author	Bart	Bart	Hanna	Giglioli	Seker	Costanzo	Marenzi	Costanzo
Year	2005	2012	2012	2011	2016	2007	2014	2015
Country	USA	USA	USA	Italy	Turkey	USA	Italy	USA
Trial	RAPID-CHF	CARRESS		ULTRADISCO		UNLOAD	CUORE	AVOID-HF
Study design	RCT	RCT	RCT	RCT	RCT	RCT	RCT	RCT
Reference	16	17	11	10	9	7	19	18
Sample size	40	188	36	30	30	200	56	221
Intervention group size	20	94	17	15	10	100	27	110
Control group size	20	94	19	15	20	100	29	111
Age (years) in UF group	67.5	66	60	72.4	66.5	62	75	67
Age (years) in UC group	69.5	69	59	65.8	66.8	63	73	67
Male (%) in UF group	70	72	84.2	87	60	70	81	69.1
Male (%) in UC group	70	78	76	87	65	68	83	73
Comorbidities								
Hypertension (%) in UF group	60		42.1	20	100	74	48	88.2
Hypertension (%) in UC group	65		52.9	60	85	74	66	83
DM (%) in UF group	35	67	36.8	40	60	50	59	61.8
DM (%) in UC group	53	65	29.4	60	50	50	45	64
Medication								
ACE/ARB (%) in UF group	70	52		86.7		63	74	38.2
ACE/ARB (%) in UC group	70	55		80		68	66	43.2
Beta blocker (%) in UF group	75	78		66.7		65	74	52.7
Beta blocker (%) in UC group	65	79		73.3		66	76	57.7
Furosemide or equivalents (%) in UF group	65	96		100		72	100	
Furosemide or equivalents (%) in UC group	95	91		100		77	97	
*UF* – ultrafiltration, *UC* – usual care, *DM* – diabetes mellitus, *ACE* – angiotensin-converting enzyme inhibitor, *ARB* – angiotensin II receptor blocker, *HF* – heart failure

**Table 2:** Outcomes of the included studies

First author	Bart	Bart	Hanna	Giglioli	Seker	Costanzo	Marenzi	Costanzo
Year	2005	2012	2012	2011	2016	2007	2014	2015
Intervention group size	20	94	17	15	10	100	27	110
Control group size	20	94	19	15	20	100	29	111
Worsening HF in UF group		31	2			39		4
Worsening HF in UC group		28	7			63		3
Renal impairment in UF group		17	8		2	21		2
Renal impairment in UC group		14	6		1	17		2
Rehospitalization for HF in UF group		23				16	1	10
Rehospitalization for HF in UC group		24				28	14	22
Deaths in UF group	1	16	4		4	9	7	17
Deaths in UC group	0	13	4		2	11	11	14
Weight loss in UF group (kg)	2.5	5.7 ± 3.9	4.7 ± 3.5			5.0 ± 3.1	7.5 ± 5.5	10.7 ± 7.2
Weight loss in UC group (kg)	1.86	5.5 ± 5.1	1.0 ± 2.5			3.1 ± 0.75	7.9 ± 9.0	10.3 ± 9.2
Fluid removal in UF group (mL)	8415	7443 ± 4329	5215 ± 3406	11086 ± 1786	7872 ± 1829	4600 ± 2600		12900
Fluid removal in UC group (mL)	5375	7082 ± 4183	2167 ± 2380	10425 ± 3002	6882 ± 4221	3300 ± 2600		8900
*UF* – ultrafiltration, *UC* – usual care, *AE* – adverse events, *RCT* – randomized controlled trial, *DM* – diabetes mellitus,* ACE* – angiotensin-converting enzyme inhibitor, *ARB* – angiotensin II receptor blocker								

Follow-up duration varied among studies and among evaluated parameters, ranging from 30 days to 12 months.

### Quality assessment

**Fig. 2:**
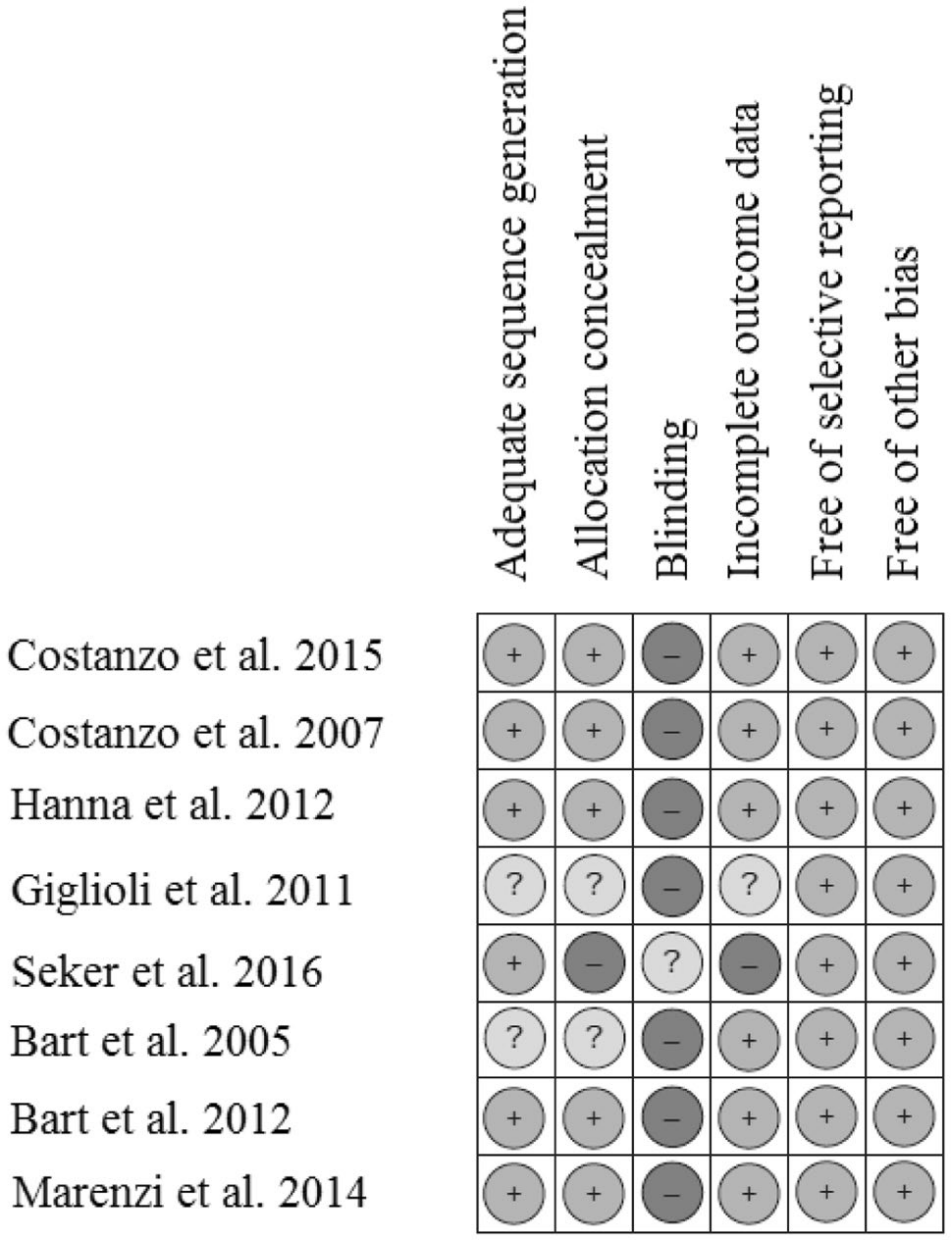
Assessment of risk of bias of included studies. Risk of bias summary according to the Cochrane Risk of Bias Tool for quality assessment of randomized controlled trials. Low risk of bias (plus-sign), unclear risk of bias (question mark) and high risk of bias (minus-sign). Short-term outcomes: fluid removal and weight loss. Long-term outcomes: adverse events, rehospitalization for heart failure (HF) and all-cause mortality

The evaluation of possible bias within the studies revealed intermediate quality. Figure 2 shows a summary of our results [13]. Notably, blinding could scarcely be assessed in the studies because of the noticeable nature of the analyzed intervention (i.e., UF). The funding of 2 included studies is a possible source of bias [7, 16].

## Outcomes

### Adverse events

#### Worsening heart failure

Four studies provided data about patients suffering from worsening heart failure during follow-up [7, 11, 17, 18]. The incidence of worsening heart failure occurred in 76 patients (34.2%) in the UF group and in 101 (45.7%) patients treated with diuretics. UF was associated with a significantly decreased risk of worsening heart failure (OR 0.632, 95% CI 0.426 to 0.936; *p* = 0.022, *I*^2^ = 67.4%).

#### Renal impairment

The incidence of renal impairment was reported in 5 studies, enrolling 675 patients in total [7, 9, 11, 17, 18]. A total of 331 patients were randomized to the UF group, while 344 received usual care treatment. Renal impairment was observed in 50 patients treated with UF and in 40 controls. We found a trend towards increased risk in usual care therapy, but this tendency failed to reach statistical significance (OR 1.386, 95% CI 0.870 to 2.209; *p* = 0.169, *I*^2^ = 0%).

#### Fluid removal

**Fig. 3:**
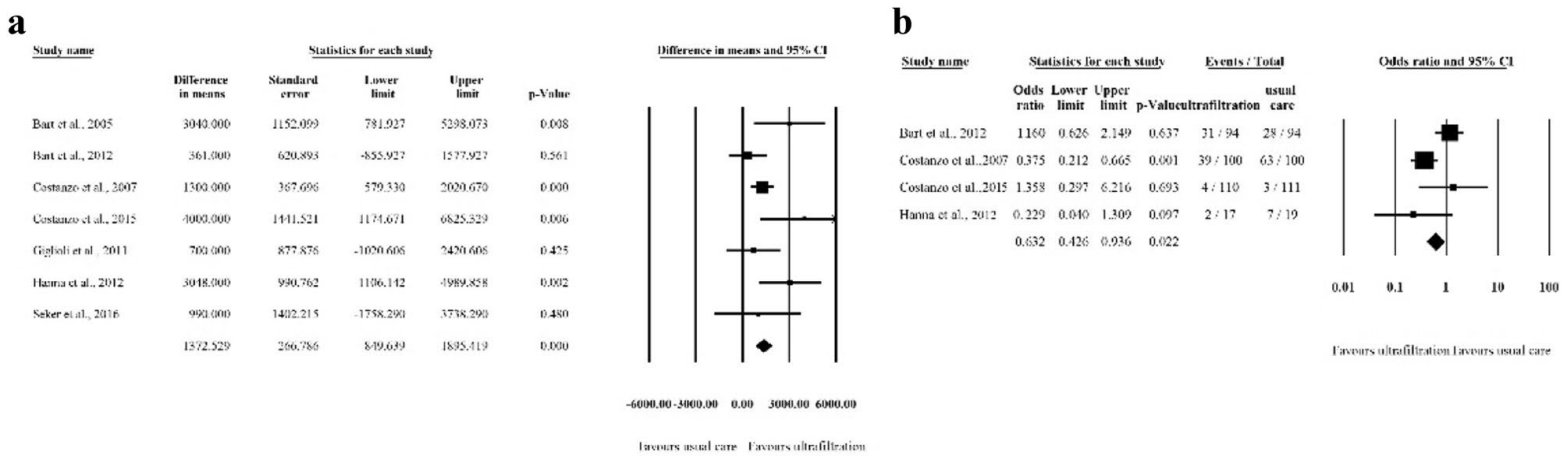
Forest plot of **a** fluid removal and **b** worsening heart failure **a**: Forest plot comparing the mean difference in fluid removal between the control and intervention groups. **b**: Forest plot comparing odds ratios of rehospitalization for heart failure. Squares represent odds ratios of rehospitalization for heart failure. Squares represent oddsratios of HF-related rehospitalization in the UF versus control group. The size of the square is proportional to the study weight. Error bars represent the 95% confidence interval. Diamonds represent pooled estimates for odds ratios with 95% CIs

Seven studies enrolling 745 patients provided data on the extent of fluid removal [7, 9–11, 17, 18]. Treatment with ultrafiltration led to a significantly larger net volume of fluid removal compared to usual care (difference in means: 1372.5 mL, 95% CI 849.6 to 1895.4 mL; *p* < 0.001, *I*^2^ = 48.41%) (Fig. 3a). The time of measurement for these outcomes ranged from 24 to 96 h, except for Marenzi, who measured fluid removal at hospital discharge, and Hanna, who measured the removal upon completion of the intervention.

#### Rehospitalization for heart failure

Heart failure-related rehospitalization was reported in 4 studies [7, 17–19]. Rehospitalization was reported for 50 out of 316 patients in the UF group and for 88 out of 320 patients randomized to the UC group. UF resulted in a significant reduction in rehospitalization (OR 0.543; 95% CI 0.362 to 0.815; *p* = 0.003, *I*^2^ = 68.4%) (Fig. 3b). The follow-up duration for evaluation of rehospitalization ranged from 30 days [7], 60 days [16], and 90 days [18] to 1 year [19].

#### Weight loss

Data on weight loss were reported in 5 studies [7, 11, 17–19] and included 741 patients in total. Weight loss in the UF group was significantly greater than weight loss in patients treated with usual care (difference in means 1.592 kg, 95% CI 1.039 to 2.144 kg; *p* < 0.001, *I*^2^ = 65.88%). Weight loss was evaluated at 24 to 96 h, except for Marenzi et al., who measured weight loss at hospital discharge, and Hanna et al., who measured weight loss at the end of the intervention period.

#### All-cause mortality

All-cause mortality was reported in 7 studies and occurred in 58 out of 378 patients in the UF group and in 57 out of 393 patients in the UC group [7, 9–11, 17–19]. Our analysis showed no difference in mortality between the two groups (OR 1.134; 95% CI 0.754 to 1.706; *p* = 0.546, *I*^2^ = 0%). The follow-up duration ranged from 30 days to 1 year.

### Risk of bias within studies

The results of the Cochrane Risk of Bias Tool for quality assessment of RCTs are displayed in Fig. 2. The industry funding of two included studies represents a possible special source of bias [7, 16].

The testing of heterogeneity across studies using Cochran’s *Q* and the *I*^2^ statistics reveals varying results for the investigated parameters. While our analysis found no evidence of heterogeneity for renal impairment (*Q* test *p* = 0.858; *I*^2^ = 0%), fluid removal (*Q* test *p* = 0.071; *I*^2^ = 48.4%), or all-cause mortality (*Q* test *p* = 0.504; *I*^2^ = 0%), there was evidence of heterogeneity for worsening HF (*Q* test *p* = 0.027; *I*^2^ = 67.4%), rehospitalization for HF (*Q* test *p* = 0.023; *I*^2^ = 68.4%), and weight loss (*Q* test *p* = 0.0195; *I*^2^ = 65.9%).

### Assessment of publication bias

**Fig. 4:**
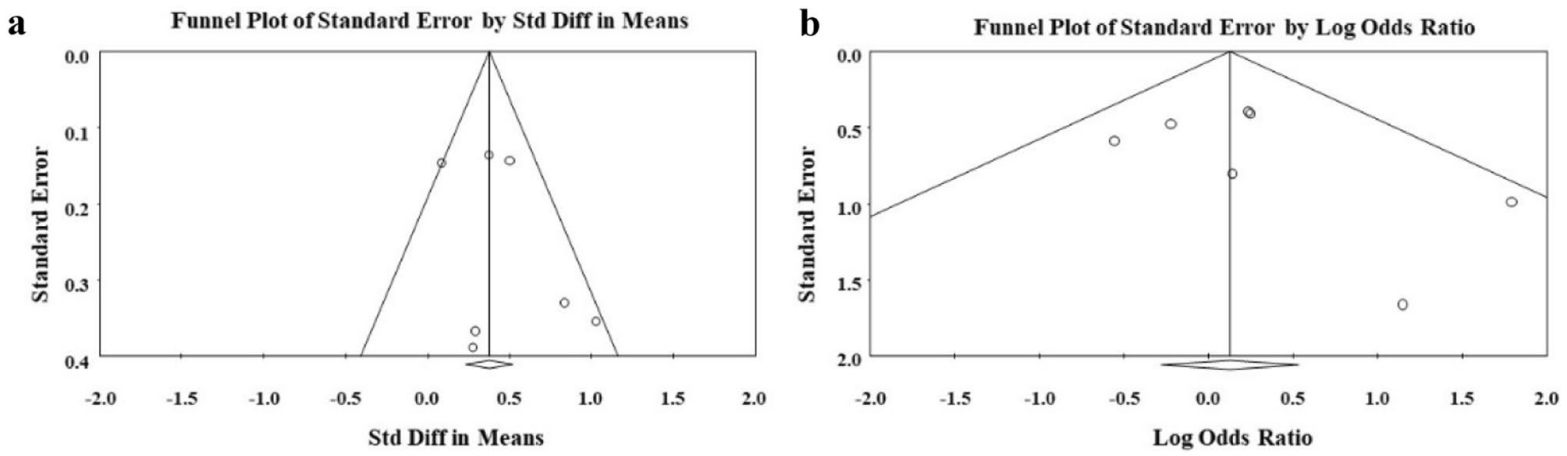
Funnel plot for visualization of publication bias across studies for fluid removal **a** and all-cause mortality **b**

Visual inspection of the funnel plots showed no evidence of publication bias for fluid removal (Fig. 4a), but visual evidence of publication bias was observed in the funnel plot for all-cause mortality (Fig. 4b).

## Discussion

In this meta-analysis of 8 studies involving 801 patients, we demonstrated the potential of UF to remove an increased volume of fluid compared with diuretic treatment, while showing no increased risk of deterioration in renal function in volume-overloaded HF patients. Moreover, UF reduces the risks of worsening HF and rehospitalization for HF.

Our findings implicate UF as a valid treatment alternative to diuretics in this patient population. The present evaluation is congruent with a previous review, which showed that UF treatment effectively removed larger volumes of fluid without significant changes in serum creatinine levels [20].

In these high-risk populations, with a considerable prevalence of cardiorenal syndrome, preservation of kidney function is an important task [21]. Patients developing renal failure and requiring dialysis exhibit a 2.7-fold increase in mortality within 12 months compared with those who do not show renal insufficiency (95% vs. 35%).[22]

Importantly, UF improves quality of life to a similar extent as diuretic treatment [18]. Bart et al. found improved results for dyspnea scores and congestive HF symptoms after UF therapy [16]. In a meta-analysis by Kabach et al., UF was associated with a reduced risk of clinical worsening and an increased likelihood for clinical decongestion; however, UF was found to have no effect on rates of rehospitalization and mortality [23]. While the meta-analysis by Kabach et al. was able to prove the advantages of UF in terms of decongestion, we were able to not only reproduce some of their findings but also emphasize the advantages of UF in preserving renal function and lowering rates of rehospitalization with the addition of two more recent RCTs.

In our present study, we showed a sustained advantage of UF treatment over usual care regarding the number of HF-related rehospitalizations between 30 days and 12 months after therapy. Long-lasting benefits of UF therapy were also described by Agostoni et al., who reported increased exercise performance that persisted for 3 months, as well as improved pulmonary function and lowered norepinephrine levels at rest that lasted up to 6 months after UF therapy in congestive HF patients.[24] While chronic diuretic treatment appears to have negative effects on neurohormonal activation, UF may contribute to the sustained benefit regarding rehospitalization by decreasing the activation of the neurohormonal axis, although the available data remain contradictory. Agostoni et al. reported a significant decrease in plasma renin activity and serum levels of norepinephrine and aldosterone after UF therapy [24]. While Giglioli et al. were able to reproduce these findings for aldosterone, Seker et al. found no difference between the groups for renin and aldosterone levels [9]. Moreover, an analysis of the CARRESS trial found higher plasma renin activity among patients treated with UF than among those in the diuretic therapy group [25].

UF might reduce the diuretic dose needed to preserve the patient’s euvolemia. Costanzo et al. described significantly lower doses needed in the UF group at 10 days after discharge than in the diuretic group.[7] Similarly, Marenzi et al. reported that the UF group needed significantly lower doses of diuretics at the 12-month follow-up [19]. The ESCAPE trial revealed a strong correlation between the in-hospital loop diuretic dose and 6-month mortality. Notably, the dose remained a significant predictor of mortality.[26] Our analysis found no influence on all-cause mortality.

It should also be mentioned that our analysis has some limitations. First, we detected considerable differences in the intervention protocols (UF rate, diuretic dosage, etc.) and time of measurement across the included studies. Thus, clinical diversity might have influenced the outcome of this analysis. Second, we found possible risks of bias within the studies. In particular, funding might have influenced the results [7, 16]. Third, our analysis found evidence of heterogeneity for certain outcomes, which might be explained by the small number of studies. Fourth, different search terms were used to identify suitable studies during the selection process. Search terms were individually adapted to the databases to obtain a high number of results.

This analysis supports the efficacy and safety of UF therapy. UF has the potential to remove a larger net volume of fluid without increasing the risk of adverse events. However, the lack of physician experience with extracorporeal therapies, the need for veno-venous access and the associated costs for the device and related disposable items are obvious disadvantages and obstacles to the widespread use of this technique. Further studies are necessary to investigate the adverse events related to UF treatment, specifically identify its potential advantages and clearly define patient populations that may benefit most from primary or early UF treatment.

## Electronic supplementary material

Below is the link to the electronic supplementary material.

Supplementary file1 (DOCX 39 KB)

## Abbreviations

UF: Ultrafiltration
ADHF: Acute decompensated heart failure
UC: Usual care
RCT: Randomized controlled trial
OR: Odds ratio
CI: Confidence interval
DM: Diabetes mellitus
ACE: Angiotensin-converting enzyme
ARB: Angiotensin II receptor blocker
NYHA: New York Heart Association
HF: Heart failure

## References

[1] Ponikowski P, Voors AA, Anker SD, Bueno H, Cleland JGF (2016). ESC Guidelines for the diagnosis and treatment of acute and chronic heart failure. Eur J Heart Fail.

[2] Krumholz HM, Parent EM, Tu N, Vaccarino V, Wang Y, Radford MJ (1997). Readmission after hospitalization for congestive heart failure among Medicare beneficiaries. Arch Intern Med.

[3] Adams KF, Fonarow GC, Emerman CL, LeJemtel TH, Costanzo MR, Abraham WT (2005). Characteristics and outcomes of patients hospitalized for heart failure in the United States: rationale, design, and preliminary observations from the first 100,000 cases in the Acute Decompensated Heart Failure National Registry (ADHERE). Am Heart J.

[4] Ross JS, Chen J, Lin Z, Bueno H, Curtis JP, Keenan PS (2010). Recent national trends in readmission rates after heart failure hospitalization. Circ Heart Fail.

[5] Ravnan SL, Ravnan MC, Deedwania PC (2002). Diuretic resistance and strategies to overcome resistance in patients with congestive heart failure. Congestive Heart Failure.

[6] Yancy CW, Jessup M, Bozkurt B, Butler J, Casey DE Jr, Drazner MH et al (2013) ACCF/AHA guideline for the management of heart failure. A report for the American College of Cardiology Foundation/American Heart Association Task Force on Practice Guidelines. Circulation 128:e240–327 10.1161/CIR.0b013e31829e8776 23741058

[7] Costanzo MR, Guglin ME, Saltzberg MT, Jessup ML, Bart BA, Teerlink JR (2007). Ultrafiltration versus intravenous diuretics for patients hospitalized for acute decompensated heart failure. J Am Coll Cardiol.

[8] Bart BA (2016). Should ultrafiltration be used preferentially instead of diuretics for the initial treatment of ADHF patients?. Circulation Heart Failure.

[9] Şeker A, Katayas M, Hüzmeli C, Candan F, Yilmaz MB (2016). Comparison of ultrafiltration and intravenous diuretic therapies in patients hospitalized for acute decompensated biventricular heart failure. Turkish Nephrology, Dialysis and Transplantation Journal.

[10] Giglioli C, Landi D, Cecchi E, Chiostri M, Gensini GF, Valente S et al (2011) Effects of ULTRAfiltration vs. DIureticS on clinical, biohumoral and haemodynamic variables in patients with deCOmpensated heart failure: the ULTRADISCO study. European Journal of Heart Failure 13:337–346 10.1093/eurjhf/hfq207 21131387

[11] Hanna MA, Tang WH, Teo BW, O'Neill JO, Weinstein DM, Lau SM (2012). Extracorporeal ultrafiltration vs conventional diuretic therapy in advanced decompensated heart failure. Congestive Heart Failure.

[12] Moher D, Liberati A, Tetzlaff J, Altman DG (2009). The PRISMA Group (2009) preferred reporting item for systematic reviews and meta-analyses: the PRISMA statement. PLoS Medicine.

[13] Higgins JP, Altman DG, Gøtzsche PC, Jüni P, Moher D, Oxman AD (2011). The Cochrane Collaboration’s tool for assessing risk of bias in randomised trials. BMJ.

[14] Mantel N, Haenszel W (1959). Statistical aspects of the analysis of data from retrospective studies of disease. J Natl Cancer Inst.

[15] Cochran WG (1954). The combination of estimates from different experiments. Biometrics.

[16] Bart BA, Boyle A, Bank AJ, Anand I, Olivari MT, Kraemer M et al (2005) Ultrafiltration versus usual care for hospitalized patients with heart failure. The Relief for Acutely Fluid-Overloaded Patients With Decompensated Congestive Heart Failure (RAPID-CHF) Trial. Journal of the American College of Cardiology 46:2043–2046 10.1016/j.jacc.2005.05.098 16325039

[17] Bart BA, Goldsmith SR, Lee KL, O'Connor CM, Givertz MM, Bull DA (2012). Ultrafiltration in Decompensated heart failure with cardiorenal syndrome. The New England Journal of Medicine.

[18] Costanzo MR, Negoianu D, Jaski BE, Bart BA, Heywood JT, Anand IS et al (2015) Aquapheresis versus intravenous diuretics and hospitalizations for heart failure. JACC: Heart Failure 4:95–105 10.1016/j.jchf.2015.08.005 26519995

[19] Marenzi G, Muratori M, Cosentino ER, Rinaldi ER, Donghi V, Milazzo V (2014). Continuous ultrafiltration for congestive heart failure: The CUORE Trial. J Cardiac Fail.

[20] Zhi Q, Liang JC (2013). Diuretics and ultrafiltration in acute heart failure syndrome: A meta-analysis. International Heart Journal.

[21] Ronco C, Cicoira M, McCullough PA et al (2012) Cardiorenal Syndrome Type 1. Pathophysiological crosstalk leading to combined heart and kidney dysfunction in the setting of acutely decompensated heart failure. Journal of the American College of Cardiology 60:1031–1042 10.1016/j.jacc.2012.01.077 22840531

[22] Wehbe E, Patarroyo M, Taliercio JJ, Starling RC, Nally JV, Tang WH (2015). Renal failure requiring dialysis complicating slow continuous ultrafiltration in acute heart failure: Importance of systolic perfusion pressure. J Cardiac Fail.

[23] Kabach M, Alkhawam H, Shah S, Joseph G, Donath EM, Moss N, Rosenstein RS (2017). Ultrafiltration versus intravenous loop diuretics in patients with acute decompensated heart failure: A meta-analysis of clinical trials. Acta Cardiol.

[24] Agostoni P, Marenzi G (2001). Sustained benefit from ultrafiltration in moderate congestive heart failure. Cardiology.

[25] Mentz RJ, Stevens SR, DeVore AD, Lala A, Vader J, AbouEzzeddine OF (2015). Decongestion strategies and renin-aldosterone system activation in acute heart failure. JACC Heart Failure.

[26] Hasselblad V, Stough WG, Shah MR, Lokhnygina Y, O'Connor CM, Califf RM (2007). Relation between dose of loop diuretics and outcomes in a heart failure population: Results of the ESCAPE trial. Eur J Heart Fail.

