# Supplementary material for: Ultrafiltration is better than diuretic therapy for volume-overloaded acute heart failure patients: a meta-analysis
Source: Heart Fail Rev. 2020 Nov 26;26(3):577–85. doi: 10.1007/s10741-020-10057-7 (PMC8024232; doi:10.1007/s10741-020-10057-7)
Supplement: Supplementary file 1 — Supplementary file1 (DOCX 39 KB) [file 10741_2020_10057_MOESM1_ESM.docx]

(10) Appendices/Online supplement

The following tables were implemented according to the risk of bias table of the Cochrane Handbook for Systematic Reviews of Interventions. The findings served as a reference for the risk of bias summary assessing the quality of the randomized controlled trials that were included in our analysis. The tables were included to elucidate the details of the underlying data that were used for the rating.

Costanzo et al. 2015

| Entry: | Judgment: | Description: |
| --- | --- | --- |
| Adequate sequence generation? | Yes. | Quote: "Using a central web-based system, study subjects were randomized to one or the other fluid removal therapies" (Costanzo 2015) |
| Allocation concealment? | Yes. |  |
| Blinding? | No. | Open-label |
| Incomplete outcome data addressed? (Short-term outcomes) | Yes. | No missing data. |
| Incomplete outcome data addressed? (Long-term outcomes) | Yes. | "By 90 days, of the 221 patients, 165 (75%) completed the study, 31 (14%) died, 9 (4%) were lost to follow-up, 3 (1.4%) withdrew consent, 7 (3%) were removed from the trial by their physician for medical reasons, and 6 (2.7%) did not finish the study due to other causes." (Costanzo 2015)  Note: Intention-to-treat was used. Reasons for dropouts were provided. |
| Free of selective reporting? | Yes. | Detailed protocol available. All outcomes are reported in the prespecified way. |
| Free of other bias? | Yes. | Note: Trial was stopped early due to slow recruitment. |

Seker et al. 2016

| Entry: | Judgment: | Description: |
| --- | --- | --- |
| Adequate sequence generation? | Yes. | Quote: "Patients hospitalized for heart failure were randomly assigned in a 1:1 ratio to either ultrafiltration therapy or diuretic therapy." (Seker 2016) |
| Allocation concealment? | No. | Note: Rotation used. |
| Blinding? | No. | Study did not provide any information about blinding. Probably not done. |
| Incomplete outcome data addressed? (Short-term outcomes) | No. | Quote: "Another nine patients had been enrolled to the Ultrafiltration group, but these 9 patients were excluded from the study as ultrafiltration therapy had been terminated because of technical reasons such as ineffective operation of the ultrafiltration device and clotting in the set." |
| Incomplete outcome data addressed? (Long-term outcomes) | No. | See short-term outcomes. |
| Free of selective reporting? | Yes. | Detailed protocol available. All outcomes are reported in the prespecified way. |
| Free of other bias? | Yes. |  |

Bart et al. 2012

| Entry: | Judgment: | Description: |
| --- | --- | --- |
| Adequate sequence generation? | Yes. | Quote: "With the use of an automated Web-based system, patients were randomly assigned, in a 1:1 ratio, to either ultrafiltration therapy or pharmacologic therapy." (Bart 2012) |
| Allocation concealment? | Yes. | Note: Web-based randomization |
| Blinding? | No. | Unblinded |
| Incomplete outcome data addressed? (Short-term outcomes) | Yes. | Note: Intention-to-treat analysis. Imputation of missing data. Reasons for missing data were provided. Missing data are balanced between groups. |
| Incomplete outcome data addressed? (Long-term outcomes) | Yes. | See short-term outcomes. |
| Free of selective reporting? | Yes. | Detailed protocol available. All outcomes are reported in the prespecified way. |
| Free of other bias? | Yes. |  |

Costanzo et al. 2007

| Entry: | Judgment: | Description: |
| --- | --- | --- |
| Adequate sequence generation? | Yes. | Quote: "Patients were randomized through a secure website." (Costanzo 2007) |
| Allocation concealment? | Yes. | Web-based randomization |
| Blinding? | No. | Unblinded |
| Incomplete outcome data addressed? (Short-term outcomes) | No. | 48 h weight loss: 17/100 missing from intervention group, 16/100 missing from control group. No reasons provided. |
| Incomplete outcome data addressed? (Long-term outcomes) | No. | 90-day rehospitalization: 11/100 missing from intervention group, 13/100 from control group. No reasons provided. |
| Free of selective reporting? | Yes. | Detailed protocol available. All outcomes are reported in the prespecified way. |
| Free of other bias? | No. | Funding by CHF Solutions |

Hanna et al. 2012

| Entry: | Judgment: | Description: |
| --- | --- | --- |
| Adequate sequence generation? | Yes. | Computer-based randomization. Block system used. |
| Allocation concealment? | Yes. | Quote: "participants were randomized in blocks of 4 and 2 within two strata based on the baseline GFR" (Hanna 2012) |
| Blinding? | No. | Open-label |
| Incomplete outcome data addressed? (Short-term outcomes) | Yes. | Intention-to-treat analysis. Withdrawals are provided with reasons. |
| Incomplete outcome data addressed? (Long-term outcomes) | Yes. | See short-term outcomes. |
| Free of selective reporting? | Yes. | Detailed protocol available. All outcomes are reported in the prespecified way, but selective underreporting: "N.S." for not significant used instead of exact p values. |
| Free of other bias? | Yes. | Quote: "An estimated 44 participants were to be enrolled in the study to achieve the 40 required for study analysis." (Hanna 2012)  Note: Just 36 patients due to "slow recruitment". |

Marenzi et al. 2014

| Entry: | Judgment: | Description: |
| --- | --- | --- |
| Adequate sequence generation? | Yes. | Randomization in a 1:1 ratio, based on computer generated random numbers |
| Allocation concealment? | Yes. | Computer generated randomization |
| Blinding? | No. | Unblinded |
| Incomplete outcome data addressed? (short-term outcomes) | Yes. | Intention-to-treat analysis was performed |
| Incomplete outcome data addressed? (Long-term outcomes) | Yes. | See short-term outcomes. |
| Free of selective reporting? | Yes. | Detailed protocol available. All outcomes are reported in the prespecified way. |
| Free of other bias? | Yes. |  |

Bart et al. 2005

| Entry: | Judgment: | Description: |
| --- | --- | --- |
| Adequate sequence generation? | Unclear. | Quote: "This was an investigator-initiated multicenter randomized controlled trial" (Bart 2005)  Note: No further information on randomization process provided. |
| Allocation concealment? | Unclear. | No information provided. |
| Blinding? | No. | No information provided.  Note: Probably not done. |
| Incomplete outcome data addressed? (Short-term outcomes) | Yes. | Intention-to-treat analysis was performed. |
| Incomplete outcome data addressed? (Long-term outcomes) | Yes. | 2 patients could not be treated, reasons were provided. Patients were balanced between the two groups. |
| Free of selective reporting? | Yes. | Detailed protocol available. All outcomes are reported in the prespecified way. |
| Free of other bias? | No. | Funding by CHF Solutions. |

Giglioli et al. 2011

| Entry: | Judgment: | Description: |
| --- | --- | --- |
| Adequate sequence generation? | Unclear. | No detailed information about randomization process provided. |
| Allocation concealment? | Unclear. | No information provided. |
| Blinding? | No. | No information provided.  Note: Probably not done. |
| Incomplete outcome data addressed? (Short-term outcomes) | Unclear. | Number of randomized patients was not provided. |
| Incomplete outcome data addressed? (Long-term outcomes) | Unclear. | See short-term outcomes. |
| Free of selective reporting? | Yes. | Detailed protocol available. All outcomes are reported in the prespecified way. |
| Free of other bias? | Yes. |  |

(11) Tables with Captions

Table I: Characteristics of randomized clinical trials.

| First author | Bart | Bart | Hanna | Giglioli | Seker | Costanzo | Marenzi | Costanzo |
| --- | --- | --- | --- | --- | --- | --- | --- | --- |
| Year | 2005 | 2012 | 2012 | 2011 | 2016 | 2007 | 2014 | 2015 |
| Country | USA | USA | USA | Italy | Turkey | USA | Italy | USA |
| Trial | RAPID-CHF | CARRESS |  | ULTRADISCO |  | UNLOAD | CUORE | AVOID-HF |
| Study design | RCT | RCT | RCT | RCT | RCT | RCT | RCT | RCT |
| Number of reference | 16 | 17 | 11 | 10 | 9 | 7 | 19 | 18 |
| Sample size | 40 | 188 | 36 | 30 | 30 | 200 | 56 | 221 |
| Intervention group size | 20 | 94 | 17 | 15 | 10 | 100 | 27 | 110 |
| Control group size | 20 | 94 | 19 | 15 | 20 | 100 | 29 | 111 |
| Age (years) in UF group | 67.5 | 66 | 60 | 72.4 | 66.5 | 62 | 75 | 67 |
| Age (years) in UC group | 69.5 | 69 | 59 | 65.8 | 66.8 | 63 | 73 | 67 |
| Male (%) in UF group | 70 | 72 | 84.2 | 87 | 60 | 70 | 81 | 69.1 |
| Male (%) in UC group | 70 | 78 | 76 | 87 | 65 | 68 | 83 | 73 |
| Comorbidities: |  |  |  |  |  |  |  |  |
| Hypertension (%) in UF group | 60 |  | 42.1 | 20 | 100 | 74 | 48 | 88.2 |
| Hypertension (%) in UC group | 65 |  | 52.9 | 60 | 85 | 74 | 66 | 83 |
| DM (%) in UF group | 35 | 67 | 36.8 | 40 | 60 | 50 | 59 | 61.8 |
| DM (%) in UC group | 53 | 65 | 29.4 | 60 | 50 | 50 | 45 | 64 |
| Medication: |  |  |  |  |  |  |  |  |
| ACE/ARB (%) in UF group | 70 | 52 |  | 86.7 |  | 63 | 74 | 38.2 |
| ACE/ARB (%) in UC group | 70 | 55 |  | 80 |  | 68 | 66 | 43.2 |
| Beta blocker (%) in UF group | 75 | 78 |  | 66.7 |  | 65 | 74 | 52.7 |
| Beta blocker (%) in UC group | 65 | 79 |  | 73.3 |  | 66 | 76 | 57.7 |
| Furosemide or equivalents (%) in UF group | 65 | 96 |  | 100 |  | 72 | 100 |  |
| Furosemide or equivalents (%) in UC group | 95 | 91 |  | 100 |  | 77 | 97 |  |

Table II: Outcomes of the included studies.

| First author | Bart | Bart | Hanna | Giglioli | Seker | Costanzo | Marenzi | Costanzo |
| --- | --- | --- | --- | --- | --- | --- | --- | --- |
| Year | 2005 | 2012 | 2012 | 2011 | 2016 | 2007 | 2014 | 2015 |
| Intervention group size | 20 | 94 | 17 | 15 | 10 | 100 | 27 | 110 |
| Control group size | 20 | 94 | 19 | 15 | 20 | 100 | 29 | 111 |
| Worsening HF in UF group |  | 31 | 2 |  |  | 39 |  | 4 |
| Worsening HF in UC group |  | 28 | 7 |  |  | 63 |  | 3 |
| Renal impairment in UF group |  | 17 | 8 |  | 2 | 21 |  | 2 |
| Renal impairment in UC group |  | 14 | 6 |  | 1 | 17 |  | 2 |
| Rehospitalization for HF in UF group |  | 23 |  |  |  | 16 | 1 | 10 |
| Rehospitalization for HF in UC group |  | 24 |  |  |  | 28 | 14 | 22 |
| Deaths in UF group | 1 | 16 | 4 |  | 4 | 9 | 7 | 17 |
| Deaths in UC group | 0 | 13 | 4 |  | 2 | 11 | 11 | 14 |
| Weight loss in UF group (kg) | 2.5 | 5.7 ± 3.9 | 4.7 ± 3.5 |  |  | 5.0 ± 3.1 | 7.5 ± 5.5 | 10.7 ± 7.2 |
| Weight loss in UC group (kg) | 1.86 | 5.5 ± 5.1 | 1.0 ± 2.5 |  |  | 3.1 ± 0.75 | 7.9 ± 9.0 | 10.3 ± 9.2 |
| Fluid removal in UF group (mL) | 8415 | 7443 ± 4329 | 5215 ± 3406 | 11086 ± 1786 | 7872 ± 1829 | 4600 ± 2600 |  | 12900 |
| Fluid removal in UC group (mL) | 5375 | 7082 ± 4183 | 2167 ± 2380 | 10425 ± 3002 | 6882 ± 4221 | 3300 ± 2600 |  | 8900 |
